# *trans*-Dichloridobis[(6-nicotinoyl-2-pyridyl-κ*N* ^6^)(3-pyridyl-κ*N*)methanone]copper(II)

**DOI:** 10.1107/S1600536811017685

**Published:** 2011-05-14

**Authors:** Hong Qiang, Fan Zhang

**Affiliations:** a Department of Chemistry, Capital Normal University, Beijing 100048, People’s Republic of China

## Abstract

In the title complex, [CuCl_2_(C_17_H_11_N_3_O_2_)_2_], the Cu^II^ ion is located on an inversion center. It exhibits a distorted octa­hedral coordination geometry defined by two chloride anions at *trans* sites and four 3-pyridyl N atoms at equatorial sites from two (6-nicotinoyl-2-pyrid­yl)(3-pyrid­yl)methanone ligands. The (6-nicotinoyl-2-pyrid­yl)(3-pyrid­yl)methanone ligand can be viewed as having two pendant 3-pyridyl rings attached to a central pyridyl skeleton *via* separate carbonyl bridges, acting in a κ^2^ *N*,*N*′-chelating mode with its 3-pyridyl N atoms bound to the Cu^II^ ion. The pendant 3-pyridyl rings make a dihedral angle of 80.76 (5)°. In the crystal, mol­ecules are linked through inter­molecular C—H⋯π and C—H⋯O inter­actions, forming a three-dimentional framework.

## Related literature

For transition metal complexes with di-pyrid-2-yl ketone, see: Papaefstathiou & Perlepes (2002 ▶); Efthymiou *et al.* (2006 ▶). For the crystal structure of an analogous Cu^II^ complex, see: Wan *et al.* (2008 ▶). For C—H⋯π inter­actions, see: Umezawa *et al.* (1998 ▶).
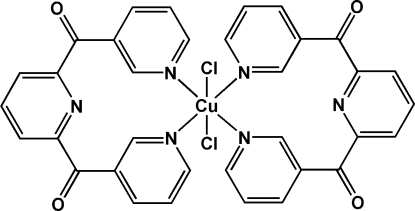


## Experimental

### 

#### Crystal data

[CuCl_2_(C_17_H_11_N_3_O_2_)_2_]
*M* *_r_* = 713.02
Monoclinic, 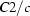

*a* = 18.728 (3) Å
*b* = 11.8971 (18) Å
*c* = 16.695 (3) Å
β = 121.522 (3)°
*V* = 3170.9 (8) Å^3^
*Z* = 4
Mo *K*α radiation
μ = 0.91 mm^−1^
*T* = 293 K
0.40 × 0.30 × 0.30 mm

#### Data collection

Bruker APEXII CCD area-detector diffractometer
Absorption correction: multi-scan (*SADABS*; Bruker, 2007 ▶) *T*_min_ = 0.848, *T*_max_ = 1.000
11215 measured reflections
3937 independent reflections
3361 reflections with *I* > 2σ(*I*)
*R*_int_ = 0.020

#### Refinement

*R*[*F* ^2^ > 2σ(*F* ^2^)] = 0.029
*wR*(*F* ^2^) = 0.084
*S* = 1.04
3937 reflections
215 parameters
H-atom parameters constrained
Δρ_max_ = 0.25 e Å^−3^
Δρ_min_ = −0.21 e Å^−3^

### 

Data collection: *APEX2* (Bruker, 2007 ▶); cell refinement: *APEX2* and *SAINT* (Bruker, 2007 ▶); data reduction: *SAINT*; program(s) used to solve structure: *SHELXS97* (Sheldrick, 2008 ▶); program(s) used to refine structure: *SHELXL97* (Sheldrick, 2008 ▶); molecular graphics: *SHELXTL* (Sheldrick, 2008 ▶); software used to prepare material for publication: *SHELXTL* and *PLATON* (Spek, 2009 ▶).

## Supplementary Material

Crystal structure: contains datablocks I, global. DOI: 10.1107/S1600536811017685/zq2102sup1.cif

Structure factors: contains datablocks I. DOI: 10.1107/S1600536811017685/zq2102Isup2.hkl

Additional supplementary materials:  crystallographic information; 3D view; checkCIF report

## Figures and Tables

**Table 1:** Hydrogen-bond geometry (Å, °) *Cg*1 is the centroid of the C13–C17,N3 ring.

*D*—H⋯*A*	*D*—H	H⋯*A*	*D*⋯*A*	*D*—H⋯*A*
C13—H13*A*⋯O1^i^	0.93	2.61	3.418 (2)	146
C2—H2*A*⋯*Cg*1^ii^	0.93	2.73	3.621 (3)	162

Symmetry codes: (i) 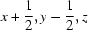; (ii) 
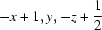
.

**Fig. 1.**
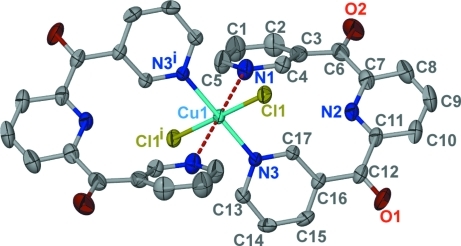
The atom-numbering scheme of the title complex (symmetry code: (i) -x+1.5, -y+0.5, -z+1.5) with red-dashed lines indicating weak Cu1-N1 bonding. Displacement ellipsoids are drawn at the 50% probability level and H atoms are omitted for clarity.

**Fig. 2.**
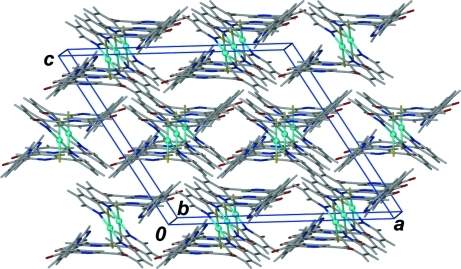
View of the crystal packing of the title compound.

## supplementary crystallographic information

### Comment

Di-pyridin-2-yl-methanone (di-pyrid-2-yl ketone, DPK), (C_5_H_4_N)_2_CO, is an extraordinarily versatile ligand among the thousands of basic building blocks that have been used in coordination chemistry and materials science (Papaefstathiou & Perlepes, 2002; Efthymiou *et al.*, 2006). Herein, we report the mononuclear Cu^II^ complex with the oligo-pyridyl ketone ligand (6-nicotinoyl-2-pyridyl)(3-pyridyl)methanone (abbreviated as L), a member of the pyridinylmethanone family.

In the crystal structure of the title complex, the center Cu^II^ adopts an octahedral coordination geometry with two chlorido depositing in trans to each other, and two 2,6-pyridinediylbis(3-pyridinyl)methanone ligands bound to the ion by four 3-pyridyl N atoms (Fig. 1). The Cu1—N3 and Cu1—Cl1 bond lengths equal 2.0412 (12) Å and 2.3087 (4) Å, respectively, while the Cu-N1 exhibits weak bonding with the Cu-N1 distance of 2.615 (1) Å. The latter Cu—N bonds are remarkably longer than that (about 2.03 Å) in the similar complex Cu(L)_2_(BF_4_)_2_ (Wan *et al.* 2008). The pendant 3-pyridyl rings exhibit a dihedral angle of 80.76 (5)°. The mononuclear complex units link each other through the intermolecular C2—H2A···π and C13—H13A···O1^ii^ interactions to form a three-dimentional framework, as shown in Fig. 2. For the C—H···π interaction (Umezawa *et al.* 1998), the C2···Cg1 distance (where Cg1 is the centroid of the ring containing N3^i^; i: -x+1, -y, 0.5-z) is 3.621 (3) Å, and the C2—H2A···Cg1 angle is 161.8° . For the C—H···O interaction, the C13···O1^ii^ distance is 3.418 (2) Å, and the C13—H13A···O1^ii^ angle is 146.2° (ii: x+0.5, y-0.5, z).

### Experimental

The (6-nicotinoyl-2-pyridyl)(3-pyridyl)methanone ligand was obtained following the reaction procedure as reported in literature (Wan *et al.*, 2008). Reaction of (6-nicotinoyl-2-pyridyl)(3-pyridyl)methanone (29 mg, 0.1 mmol) with CuCl_2_ (7 mg, 0.05 mmol) in acetonitrile formed *trans*-[Cu(C_17_H_11_N_3_O_2_)_2_Cl_2_] as a blue solution, which was left stand in air for four days to obtain block-like crystals (yield 13.1mg, 61%).

### Refinement

The hydrogen atoms were placed in idealized positions and allowed to ride on the relevant carbon atoms, with C—H = 0.93 Å and *U*_iso_(H) = 1.2*U*_eq_(C).

### Crystal data

**Table:**

[CuCl_2_(C_17_H_11_N_3_O_2_)_2_]	*Z* = 4
*M**_r_* = 713.02	*F*(000) = 1452
Monoclinic, *C*2/*c*	*D*_x_ = 1.494 Mg m^−^^3^
Hall symbol: -C 2yc	Mo *K*α radiation, λ = 0.71073 Å
*a* = 18.728 (3) Å	µ = 0.91 mm^−^^1^
*b* = 11.8971 (18) Å	*T* = 293 K
*c* = 16.695 (3) Å	Block, blue
β = 121.522 (3)°	0.40 × 0.30 × 0.30 mm
*V* = 3170.9 (8) Å^3^	

### Data collection

**Table:**

Bruker APEXII CCD area-detector diffractometer	3937 independent reflections
Radiation source: fine-focus sealed tube	3361 reflections with *I* > 2σ(*I*)
graphite	*R*_int_ = 0.020
ω scans	θ_max_ = 28.3°, θ_min_ = 2.1°
Absorption correction: multi-scan (*SADABS*; Bruker, 2007)	*h* = −24→24
*T*_min_ = 0.848, *T*_max_ = 1.000	*k* = −15→15
11215 measured reflections	*l* = −19→22

### Refinement

**Table:**

Refinement on *F*^2^	Secondary atom site location: difference Fourier map
Least-squares matrix: full	Hydrogen site location: inferred from neighbouring sites
*R*[*F*^2^ > 2σ(*F*^2^)] = 0.029	H-atom parameters constrained
*wR*(*F*^2^) = 0.084	*w* = 1/[σ^2^(*F*_o_^2^) + (0.0423*P*)^2^ + 2.1803*P*] where *P* = (*F*_o_^2^ + 2*F*_c_^2^)/3
*S* = 1.04	(Δ/σ)_max_ = 0.001
3937 reflections	Δρ_max_ = 0.25 e Å^−^^3^
215 parameters	Δρ_min_ = −0.21 e Å^−^^3^
0 restraints	Extinction correction: *SHELXL97* (Sheldrick, 2008), Fc^*^=kFc[1+0.001xFc^2^λ^3^/sin(2θ)]^-1/4^
Primary atom site location: structure-invariant direct methods	Extinction coefficient: 0.00079 (17)

### Special details

**Table:**

Geometry. All esds (except the esd in the dihedral angle between two l.s. planes) are estimated using the full covariance matrix. The cell esds are taken into account individually in the estimation of esds in distances, angles and torsion angles; correlations between esds in cell parameters are only used when they are defined by crystal symmetry. An approximate (isotropic) treatment of cell esds is used for estimating esds involving l.s. planes.
Refinement. Refinement of F^2^ against ALL reflections. The weighted R-factor wR and goodness of fit S are based on F^2^, conventional R-factors R are based on F, with F set to zero for negative F^2^. The threshold expression of F^2^ > 2sigma(F^2^) is used only for calculating R-factors(gt) etc. and is not relevant to the choice of reflections for refinement. R-factors based on F^2^ are statistically about twice as large as those based on F, and R- factors based on ALL data will be even larger.

### Fractional atomic coordinates and isotropic or equivalent isotropic displacement parameters (Å^2^)

**Table:**

	*x*	*y*	*z*	*U*_iso_*/*U*_eq_	
Cu1	0.7500	0.2500	0.5000	0.03142 (9)	
Cl1	0.69747 (3)	0.11938 (3)	0.38011 (3)	0.03905 (11)	
O2	0.77486 (11)	0.74717 (10)	0.35782 (15)	0.0670 (5)	
C12	0.73237 (11)	0.67642 (13)	0.36615 (13)	0.0423 (4)	
C1	0.45389 (14)	0.2790 (2)	0.33217 (19)	0.0666 (6)	
H1A	0.4120	0.2267	0.2971	0.080*	
C2	0.43565 (12)	0.39132 (18)	0.32729 (16)	0.0588 (5)	
H2A	0.3811	0.4166	0.2878	0.071*	
C3	0.49909 (10)	0.46676 (15)	0.38157 (12)	0.0404 (4)	
C4	0.57867 (10)	0.42349 (14)	0.44252 (12)	0.0386 (3)	
H4A	0.6207	0.4729	0.4830	0.046*	
N1	0.59789 (9)	0.31499 (12)	0.44589 (11)	0.0464 (3)	
C5	0.53567 (13)	0.24547 (15)	0.39018 (18)	0.0571 (5)	
H5A	0.5483	0.1698	0.3905	0.069*	
C6	0.47982 (11)	0.58898 (16)	0.37172 (13)	0.0453 (4)	
O1	0.41059 (9)	0.62239 (13)	0.35012 (13)	0.0676 (4)	
C7	0.54433 (11)	0.67304 (14)	0.38234 (13)	0.0432 (4)	
C8	0.53320 (14)	0.78598 (18)	0.39550 (17)	0.0602 (5)	
H8A	0.4891	0.8084	0.4023	0.072*	
C9	0.58907 (15)	0.86377 (16)	0.39825 (18)	0.0657 (6)	
H9A	0.5826	0.9397	0.4062	0.079*	
C10	0.65408 (13)	0.82833 (15)	0.38923 (14)	0.0520 (5)	
H10A	0.6924	0.8795	0.3908	0.062*	
C11	0.66154 (11)	0.71359 (13)	0.37759 (12)	0.0394 (4)	
N2	0.60753 (9)	0.63678 (11)	0.37358 (10)	0.0378 (3)	
N3	0.76337 (8)	0.36519 (10)	0.41813 (9)	0.0300 (3)	
C14	0.74131 (10)	0.47290 (12)	0.41611 (11)	0.0314 (3)	
H14A	0.7177	0.4935	0.4510	0.038*	
C15	0.75214 (10)	0.55503 (12)	0.36437 (11)	0.0338 (3)	
C16	0.78887 (11)	0.52392 (15)	0.31374 (12)	0.0419 (4)	
H16A	0.7983	0.5770	0.2794	0.050*	
C17	0.81099 (11)	0.41376 (15)	0.31523 (13)	0.0431 (4)	
H17A	0.8353	0.3914	0.2815	0.052*	
C13	0.79703 (10)	0.33587 (14)	0.36711 (11)	0.0368 (3)	
H13A	0.8113	0.2611	0.3667	0.044*	

### Atomic displacement parameters (Å^2^)

**Table:**

	*U*^11^	*U*^22^	*U*^33^	*U*^12^	*U*^13^	*U*^23^
Cu1	0.04854 (17)	0.01975 (13)	0.03224 (15)	−0.00100 (10)	0.02548 (13)	0.00062 (9)
Cl1	0.0554 (2)	0.02824 (18)	0.0347 (2)	−0.00295 (15)	0.02439 (18)	−0.00262 (14)
O2	0.0755 (10)	0.0343 (7)	0.1049 (14)	−0.0040 (6)	0.0566 (10)	0.0174 (7)
C12	0.0487 (9)	0.0296 (7)	0.0459 (9)	−0.0008 (7)	0.0230 (8)	0.0109 (7)
C1	0.0446 (11)	0.0549 (12)	0.0815 (16)	−0.0138 (9)	0.0198 (11)	−0.0136 (11)
C2	0.0344 (9)	0.0595 (12)	0.0661 (13)	0.0007 (8)	0.0149 (9)	−0.0019 (10)
C3	0.0366 (8)	0.0426 (9)	0.0436 (9)	0.0033 (7)	0.0221 (7)	0.0027 (7)
C4	0.0371 (8)	0.0365 (8)	0.0390 (8)	−0.0007 (6)	0.0177 (7)	0.0016 (6)
N1	0.0412 (8)	0.0358 (7)	0.0534 (9)	0.0007 (6)	0.0186 (7)	0.0041 (6)
C5	0.0498 (11)	0.0376 (10)	0.0752 (15)	−0.0054 (8)	0.0266 (11)	−0.0042 (9)
C6	0.0433 (9)	0.0466 (9)	0.0468 (10)	0.0112 (8)	0.0242 (8)	0.0031 (8)
O1	0.0517 (8)	0.0637 (9)	0.0945 (12)	0.0184 (7)	0.0432 (9)	0.0034 (8)
C7	0.0444 (9)	0.0361 (8)	0.0428 (9)	0.0093 (7)	0.0185 (8)	0.0022 (7)
C8	0.0609 (13)	0.0440 (10)	0.0716 (14)	0.0169 (9)	0.0318 (11)	−0.0033 (10)
C9	0.0750 (15)	0.0305 (9)	0.0796 (15)	0.0102 (9)	0.0320 (13)	−0.0070 (9)
C10	0.0630 (12)	0.0273 (8)	0.0548 (11)	−0.0004 (8)	0.0233 (10)	0.0011 (7)
C11	0.0489 (9)	0.0264 (7)	0.0365 (8)	0.0033 (6)	0.0178 (7)	0.0052 (6)
N2	0.0430 (7)	0.0279 (6)	0.0397 (7)	0.0055 (5)	0.0196 (6)	0.0041 (5)
N3	0.0373 (7)	0.0248 (5)	0.0326 (6)	0.0011 (5)	0.0214 (6)	0.0023 (5)
C14	0.0374 (7)	0.0264 (7)	0.0343 (7)	0.0021 (6)	0.0213 (6)	0.0036 (6)
C15	0.0366 (8)	0.0284 (7)	0.0352 (8)	−0.0024 (6)	0.0179 (6)	0.0052 (6)
C16	0.0462 (9)	0.0437 (9)	0.0407 (9)	−0.0072 (7)	0.0262 (8)	0.0073 (7)
C17	0.0491 (10)	0.0495 (9)	0.0438 (9)	−0.0021 (8)	0.0335 (8)	−0.0011 (7)
C13	0.0428 (9)	0.0332 (7)	0.0394 (8)	0.0019 (6)	0.0251 (7)	−0.0011 (6)

### Geometric parameters (Å, °)

**Table:**

Cu1—N3	2.0412 (12)	C7—N2	1.338 (2)
Cu1—N3^i^	2.0412 (12)	C7—C8	1.395 (3)
Cu1—Cl1	2.3087 (4)	C8—C9	1.380 (3)
Cu1—Cl1^i^	2.3087 (4)	C8—H8A	0.9300
O2—C12	1.215 (2)	C9—C10	1.369 (3)
C12—C15	1.495 (2)	C9—H9A	0.9300
C12—C11	1.502 (3)	C10—C11	1.396 (2)
C1—C2	1.371 (3)	C10—H10A	0.9300
C1—C5	1.376 (3)	C11—N2	1.339 (2)
C1—H1A	0.9300	N3—C14	1.3415 (18)
C2—C3	1.383 (3)	N3—C13	1.3438 (19)
C2—H2A	0.9300	C14—C15	1.3875 (19)
C3—C4	1.391 (2)	C14—H14A	0.9300
C3—C6	1.487 (2)	C15—C16	1.390 (2)
C4—N1	1.333 (2)	C16—C17	1.371 (2)
C4—H4A	0.9300	C16—H16A	0.9300
N1—C5	1.332 (2)	C17—C13	1.383 (2)
C5—H5A	0.9300	C17—H17A	0.9300
C6—O1	1.217 (2)	C13—H13A	0.9300
C6—C7	1.506 (3)		
			
N3—Cu1—N3^i^	180.0	C9—C8—C7	118.57 (19)
N3—Cu1—Cl1	90.99 (4)	C9—C8—H8A	120.7
N3^i^—Cu1—Cl1	89.01 (4)	C7—C8—H8A	120.7
N3—Cu1—Cl1^i^	89.01 (4)	C10—C9—C8	119.50 (18)
N3^i^—Cu1—Cl1^i^	90.99 (4)	C10—C9—H9A	120.2
Cl1—Cu1—Cl1^i^	180.0	C8—C9—H9A	120.2
O2—C12—C15	118.88 (17)	C9—C10—C11	118.42 (19)
O2—C12—C11	119.02 (16)	C9—C10—H10A	120.8
C15—C12—C11	122.10 (14)	C11—C10—H10A	120.8
C2—C1—C5	118.40 (19)	N2—C11—C10	123.12 (17)
C2—C1—H1A	120.8	N2—C11—C12	119.14 (14)
C5—C1—H1A	120.8	C10—C11—C12	117.71 (16)
C1—C2—C3	119.45 (18)	C7—N2—C11	117.58 (14)
C1—C2—H2A	120.3	C14—N3—C13	118.19 (13)
C3—C2—H2A	120.3	C14—N3—Cu1	120.94 (10)
C2—C3—C4	117.64 (17)	C13—N3—Cu1	120.84 (10)
C2—C3—C6	119.12 (16)	N3—C14—C15	123.02 (14)
C4—C3—C6	123.23 (16)	N3—C14—H14A	118.5
N1—C4—C3	123.46 (16)	C15—C14—H14A	118.5
N1—C4—H4A	118.3	C14—C15—C16	118.09 (14)
C3—C4—H4A	118.3	C14—C15—C12	123.33 (14)
C5—N1—C4	116.97 (16)	C16—C15—C12	118.41 (14)
N1—C5—C1	123.86 (18)	C17—C16—C15	118.97 (14)
N1—C5—H5A	118.1	C17—C16—H16A	120.5
C1—C5—H5A	118.1	C15—C16—H16A	120.5
O1—C6—C3	120.78 (18)	C16—C17—C13	119.84 (15)
O1—C6—C7	118.89 (17)	C16—C17—H17A	120.1
C3—C6—C7	120.22 (15)	C13—C17—H17A	120.1
N2—C7—C8	122.80 (18)	N3—C13—C17	121.86 (15)
N2—C7—C6	118.31 (15)	N3—C13—H13A	119.1
C8—C7—C6	118.79 (17)	C17—C13—H13A	119.1
			
C5—C1—C2—C3	−1.2 (4)	O2—C12—C11—C10	−7.4 (3)
C1—C2—C3—C4	−2.9 (3)	C15—C12—C11—C10	172.79 (16)
C1—C2—C3—C6	176.4 (2)	C8—C7—N2—C11	−0.2 (3)
C2—C3—C4—N1	5.2 (3)	C6—C7—N2—C11	176.08 (15)
C6—C3—C4—N1	−174.07 (17)	C10—C11—N2—C7	−0.8 (3)
C3—C4—N1—C5	−3.0 (3)	C12—C11—N2—C7	−178.81 (15)
C4—N1—C5—C1	−1.6 (3)	Cl1—Cu1—N3—C14	136.92 (12)
C2—C1—C5—N1	3.7 (4)	Cl1^i^—Cu1—N3—C14	−43.08 (12)
C2—C3—C6—O1	30.2 (3)	Cl1—Cu1—N3—C13	−45.23 (12)
C4—C3—C6—O1	−150.5 (2)	Cl1^i^—Cu1—N3—C13	134.77 (12)
C2—C3—C6—C7	−145.85 (19)	C13—N3—C14—C15	−0.4 (2)
C4—C3—C6—C7	33.5 (3)	Cu1—N3—C14—C15	177.50 (11)
O1—C6—C7—N2	−157.20 (19)	N3—C14—C15—C16	−1.1 (2)
C3—C6—C7—N2	18.9 (3)	N3—C14—C15—C12	−176.31 (15)
O1—C6—C7—C8	19.2 (3)	O2—C12—C15—C14	146.67 (19)
C3—C6—C7—C8	−164.64 (18)	C11—C12—C15—C14	−33.5 (2)
N2—C7—C8—C9	1.0 (3)	O2—C12—C15—C16	−28.5 (3)
C6—C7—C8—C9	−175.3 (2)	C11—C12—C15—C16	151.27 (16)
C7—C8—C9—C10	−0.8 (4)	C14—C15—C16—C17	1.5 (2)
C8—C9—C10—C11	−0.1 (3)	C12—C15—C16—C17	176.90 (16)
C9—C10—C11—N2	0.9 (3)	C15—C16—C17—C13	−0.4 (3)
C9—C10—C11—C12	178.98 (19)	C14—N3—C13—C17	1.6 (2)
O2—C12—C11—N2	170.73 (18)	Cu1—N3—C13—C17	−176.35 (13)
C15—C12—C11—N2	−9.1 (2)	C16—C17—C13—N3	−1.2 (3)

Symmetry codes: (i) −*x*+3/2, −*y*+1/2, −*z*+1.

### Hydrogen-bond geometry (Å, °)

**Table:**

Cg1 is the centroid of the C13–C17,N3 ring.

**Table:**

*D*—H···*A*	*D*—H	H···*A*	*D*···*A*	*D*—H···*A*
C13—H13A···O1^ii^	0.93	2.61	3.418 (2)	146
C2—H2A···Cg1^iii^	0.93	2.73	3.621 (3)	162

Symmetry codes: (ii) *x*+1/2, *y*−1/2, *z*; (iii) −*x*+1, *y*, −*z*+1/2.
